# Risk Factors of Neural Tube Defects in Northern Iran

**DOI:** 10.5812/ircmj.7940

**Published:** 2014-06-05

**Authors:** Mohammad Jafar Golalipour, Mostafa Qorbani, Arezo Mirfazeli, Elham Mobasheri

**Affiliations:** 1 Gorgan Congenital Malformations Research Center, Golestan University of Medical Sciences, Gorgan, IR Iran; 2 Department of Public Health, Alborz University of Medical Sciences, Karaj, IR Iran; 3 Non-Communicable Diseases Research Center, Endocrinology and Metabolism Population Sciences Institute, Tehran University of Medical Sciences, Tehran, IR Iran; 4 Department of Pediatrics, Gorgan Congenital Malformations Research Center, Golestan University of Medical Sciences, Gorgan, IR Iran; 5 Department of Obstetrics and Gynecology, Gorgan Congenital Malformations Research Center, Golestan University of Medical Sciences, Gorgan, IR Iran

**Keywords:** Neural Tube Defects, Ethnicity, Body Mass Index, Nutritional Sciences

## Abstract

**Background:**

Neural tube defects (NTDs) including spina bifida and anencephaly are the second most common birth defects with 2.8 per 1000 births in northern Iran.

**Objectives:**

This study was conducted to determine the risk factors of neural tube defects in Gorgan, north of Iran.

**Patients and Methods:**

This hospital-based, case-control study was carried out on all NTD-affected pregnancies (n = 59) during February 2007 - August 2010, and 160 healthy pregnancies were selected via convenient sampling method in three hospitals in Gorgan, north of Iran. Risk factors including maternal body mass index (BMI), season of birth, gender of the newborn, mother’s age, ethnicity, consanguineous marriage, folic acid consumption, nutrition, habitat, and education, were assessed through interviews with mothers. Univariate and multivariate logistic regression analyses were used to estimate the risks by odds ratios (ORs) and 95% confidence intervals.

**Results:**

The multivariate analysis showed that maternal BMI (normal/underweight OR: 0.23, overweight/underweight OR: 0.15, obese/underweight OR: 0.13) and maternal ethnicity (Fars/Sistani OR: 3.49) and maternal nutrition (good/poor OR: 0.46) were significantly correlated with NTDs in the newborns.

**Conclusions:**

This study showed that maternal ethnicity, insufficient nutrition, and BMI, were the main risk factors of NTDs in northern Iran.

## 1. Background

Neural tube defects (NTDs) are a group of congenital malformations including spina bifida, anencephaly, and encephalocele, which arise during the process of neurulation between the third and fourth weeks of human pregnancy (1, 2). NTDs contribute to miscarriage, infant mortality, and serious disability (3). NTDs are caused by partial or complete failure of fusion in the cranial and spinal regions of the neural tube during early embryogenesis (4). NTDs are the second most common birth defects with incidence rate of 0.97 per 1000 child births in European contraries (4). Multifactorial disturbances in embryonic neurulation have been identified as causes of NTDs (5). Incidence of different NTDs varies according to geographic conditions, race/ethnicity (5, 6), sex of the newborn (6, 7), as well as high caffeine intake, low calorie diet, alcohol consumption (4), lack of folate supplementation at any time of pregnancy (4, 6, 7), oral contraceptive usage, and passive smoking (8). In addition, other factors associated with one or both NTDs include maternal birthplace, parity, timing of prenatal care initiation, socio-economic status (9), as well as maternal age (7, 9), education, nutritional status, drug usage, presence of maternal chronic diseases, and acute infections during pregnancy (10). A previous study showed that incidence rate of NTDs is 2.88 per 1000 in northern Iran (3).

## 2. Objectives

There was no documented study regarding the risk factors of NTDs in this area; therefore, this study was conducted to determine the risk factors of NTDs in Gorgan, north of Iran.

## 3. Patients and Methods

This hospital-based, case-control study was carried out on all NTD-affected pregnancies (n: 59) during February 2007 - August 2010, and 160 healthy pregnancies were selected via convenient sampling method from three hospitals of Gorgan, north of Iran.

Ethical approval for the study was obtained from the Ethics Committee of Golestan University of Medical Sciences. The mothers’ consents were obtained for the study, along with a clearance from the institutional ethical committee. The subjects were chosen from three hospitals (Masoud, Falsafi, and Dezyani) of Gorgan, north of Iran.

Newborns with NTDs after confirmation by a pediatrician (neonatologist) as case group, normal newborns as control group, and their mothers, were evaluated. For every case, we selected the subsequent two or three healthy newborns as control group. NTDs were defined according to the International Classification of Diseases, tenth revision (ICD-10).

Dezyani Hospital is the largest specialized obstetrics and gynecology referral hospital (120 beds) in the city, with annual rate of more than 6000 deliveries. Patients are usually from moderate-to-low socioeconomic class families of various ethnic backgrounds. Masoud (60 beds) and Falsafi (100 beds) are two private general hospitals with annual rate of more than 1500 deliveries. In Gorgan, capital of Golestan province in north of Iran, three main ethnic groups are Fars, Turkman, and Sistani. The region has a population of about 350,000. Native Fars is the predominant inhabitant with the most members, Turkman is the ethnic group emigrated from central Asia more than three centuries ago, and the Sistani group emigrated from southeastern Iran half a century ago (3, 5).

Risk factors including maternal BMI, season of birth, gender of the newborn, mother’s age, ethnicity, consanguinity marriage, folic acid consumption, nutritional habitat, and education level, were assessed through interviews with mothers and recorded in a questionnaire for each mother in the case and control groups. BMIs ˂ 18, 18-24.99, 25-30, and > 30, were considered as underweight, normal, overweight and obesity, respectively (3, 5, 10).

### 3.1. Statistical Analysis

Data analysis was performed using SPSS version 16. To investigate the NTD-affecting factors, logistic regression model was used to measure the crude odds ratio (OR) of NTD occurrence for each of the independent variables. Multiple Logistic Regression (MLR) by backwards method was used to control the confounders; the independent variables with P value < 0.2 in the univariate analysis were entered in the MLR model. The results are expressed as OR with 95% confidence interval (CI). Significance level was adjusted as < 0.05.

## 4. Results

In the case group, 32 (54.2%) affected newborns were male and 27 (45.8%) were female; parents of 33 (55.9%) and 26 (44.1%) affected newborns lived in rural and urban areas, respectively. In the control group, 77 (48.1%) newborns were male and 93 (51.9%) were female; parents of 96 (60.4%) and 63 (39.6%) control newborns lived in rural and urban areas, respectively (Table 1).

The results of univariate analysis of maternal characteristics and NTD risk factors are depicted in Table 1. Based on our results, maternal age≥ 35 (OR = 1.73, CI 95%: 0.51-5.81), folate supplementation (OR = 1.13, CI 95%: 0.62-2.06), gender of newborn (OR = 1.19, CI 95%: 0.65-2.17), consanguineous marriage (OR = 1.1, CI 95%: 0.56-2.13), residency (OR = 1.2, CI 95%: 0.65-2.19), birth in summer (OR = 0.9, CI 95%: 0.4-2.03), diploma/illiterate mother (OR = 1.89, CI95 %: 0.54-6.51), gravidity > 3/prime gravid (OR = 0.71, CI 95%: 0.21-2.34), and history of abortion (OR = 1.52, CI 95%: 0.7-3.29) were not significantly associated with NTDs as risk factors.

Multivariate analysis showed that maternal BMI (normal/underweight OR: 0.23, overweight/underweight OR: 0.15, obese/underweight OR: 0.13), maternal ethnicity (Fars/Sistani OR: 3.49) and maternal nutrition (good/poor OR: 0.46) were significantly correlated with NTDs (Table 2).

**Table 1.** Univariate Models of the Association Between Maternal Risk Factors and Neural Tube Defects Occurrence ^a,b^

Risk Factors	Case	Control	OR	CI (95%)	P Value
**BMI**					
Underweight	8 (15.4)	6 (4.1)	1	-	-
Normal	23 (44.2)	66 (45.5)	0.26	0.08-0.83	0.02
Overweight	11 (21.2)	42 (29)	0.19	0.05-0.68	0.01
Obese	10 (19.2)	31 (21.4)	0.24	0.06-0.86	0.02
**Season of birth**					
Spring	13 (22)	29 (18.1)	1	-	-
Sumer	23 (39)	57 (35.6)	0.9	0.4-2.03	0.8
Autumn	7 (11.9)	32 (20)	0.48	0.17-1.39	0.17
Winter	16 (27.1)	42 (26.2)	0.85	0.35-2.03	0.71
**Consanguineous marriage**					
Yes	17 (28.8)	43 (26.9)	1.1	0.56-2.13	0.77
No	42 (71.2)	117 (73.1)	1	-	-
**Maternal age**					
≤ 20	16 (27.1)	37 (23.1)	1	-	-
20-34	37 (62.7)	115 (71.9)	0.74	0.37-1.48	0.4
≥ 35	6 (10.2)	8 (5)	1.73	0.51-5.81	0.37
**Folic acid consumption**					
Yes	28 (47.5)	71 (44.4)	1.13	0.62-2.06	0.68
No	31 (52.5)	89 (55.6)	-	-	-
**Nutrition**					
Poor	33 (55.9)	67 (42.9)	1	-	-
Good	26 (44.1)	89 (57.1)	0.59	0.32-1.08	0.09
**Habitat**					
Rural	33 (55.9)	96 (60.4)	1	-	-
Urban	26 (44.1)	63 (39.6)	1.2	0.65-2.19	0.55
**Sex**					
Male	31 (52.5)	77 (48.1)	1.19	0.65-2.17	0.56
Female	28 (47.5)	83 (51.9)	1	-	-
**Ethnicity**					
Sistani	15 (25.4)	59 (36.9)	1	-	-
Turkman	6 (10.2)	31 (19.4)	0.76	0.26-2.15	0.6
Fars	38(64.4)	70 (43.8)	2.13	1.07-4.26	0.03
**Education**					
Illiterate	4 (7.1)	20 (13)	1	-	-
Under diploma	32 (57.1)	90 (58.4)	1.77	0.56-5.59	0.32
Diploma	14 (25)	37 (24)	1.89	0.54-6.51	0.31
Higher education	6 (10.7)	7 (4.5)	4.28	0.92-19.79	0.06
**Gravidity**					
1	30 (50.8)	75 (46.9)	1	-	-
2	15 (25.4)	42 (26.2)	0.89	0.43-1.84	0.76
3	10 (16.9)	29 (18.1)	0.86	0.37-1.98	0.72
> 3	4 (6.8)	14 (8.8)	0.71	0.21-2.34	0.57
**Abortion history**					
No	47 (79.7)	137 (85.6)	1	-	-
Yes	12 (20.3)	23 (14.4)	1.52	0.7-3.29	0.28

^a^ Abbreviations: BMI, body mass index; CI, confidence interval; OR, odds ratio.

^b^ Data are presented as No. (%).

**Table 2.** Multivariate Models of the Association Between Maternal Risk Factors and Neural Tube Defects Occurrence ^a^

Risk Factor	OR	CI (95%)	P Value
**BMI**			
Underweight	1	-	-
Normal	0.22	0.06-0.81	0.02
Overweight	0.15	0.04-0.6	0.007
Obesity	0.13	0.03-0.55	0.006
**Mother’s ethnicity**			
Sistani	1	-	-
Turkman	1.36	0.4-4.62	0.61
Fars	3.49	1.48-8.19	0.004
**Nutrition**			
Poor	1	-	-
Good	0.46	0.22-0.94	0.03

^a^ Abbreviations: CI, confidence interval; OR, odds ratio.

## 5. Discussion

The rate of NTDs in different ethnic groups showed that NTDs risk in native Fars was 3.49 times more than Sistani ethnic group. Other researchers such as Canfield et al. (9) showed that anencephaly and spina bifida rates were higher among Hispanics than non-Hispanic whites and blacks. In addition, Velie et al. (11) suggested that race and ethnicity might be affecting factors in the rate of NTDs.

Regarding gender, our results indicated that the NTDs rate was higher in males than females, but our results were in contrast with other studies in southwest Iran (12) and China (8). Yin’s study in china showed that the overall sex ratio (SR) of NTD was M/F = 0.59; anencephaly, spina bifida, and encephalocele were more common in females than males, with SRs of 0.40, 0.72, and 0.82, respectively (8).

Our study showed that mother’s nutrition is significantly associated with NTD. This result was similar to Mandiracioglu’s study (10), reporting that mothers poorly nourished during pregnancy were more prevalent among the case group (OR = 4.89, CI 95%: 2.84-8.42, P = 0.000). In De Marco’s study in Italy (4), high caffeine intake (OR = 10.82, 95% CI: 3.78–31), low-calorie diet (OR = 5.15, 95% CI: 1.79–14), and occasional consumption of fruits and vegetables (OR = 3.38, 95% CI: 1.67–6.82) were the main spina bifida risk factors in the multivariate analysis.

This study showed that there was no significant difference between mothers’ ages and NTDs, whereas maternal age of over 40 years in Texas (9) and maternal age of over 30 years in Russia (13) were associated with NTDs. In Turkey (14) and Italy (4), significant association between mothers’ ages and NTDs was found.

In this study, a nonsignificant association was observed between seasonal variation and NTDs. The incidence rate of NTDs was higher in the summer, but in Nili’s study in Tehran, birth in spring and summer was significantly associated with NTDs (7). Furthermore, Obeidat and Amarin’s study in Jordan showed that more NTD-affected babies were conceived in the late summer and early autumn and concluded that seasonality affected the incidence of NTDs in the north of Jordan (15).

Regarding our results, there was no significant relation between consanguineous marriage of parents and NTDs. Various reports have attributed higher incidence of NTDs to consanguinity (16-19). Murshid in Saudi Arabia (16) reported that consanguinity of parents was found in 89% of the spina bifida parents and only 67% of the controls (P < 0.0005). A study in India (17) reported that NTD was significantly higher among babies born to parents of consanguineous marriages (P < 0.01). Furthermore, a study in Iran has shown that consanguineous marriages of all types were related with increased congenital malformations (with ratio of 43/1000 for consanguineous marriages and 28/1000 for nonconsanguineous ones) (18). A study in Iraq reported that 63.6% of NTD cases were results of consanguineous marriages (19). The possibility that consanguinity could be a risk factor for NTDs in Iranian population requires further investigations.

In this study, 55.9% and 44.1% of parents with affected newborns lived in rural and urban areas, respectively. This result was similar to a report from Texas (20). According to Luben’s study in Texas, there was no evidence that urban or rural residency was associated with changes in the rate of anencephaly or spina bifida without anencephaly in unadjusted or adjusted analyses.

In our findings, although 47.5% of women with affected newborns did not intake folic acid periconceptionally, this difference was not significant. Similar to our result, Yin’s study in China (8) reported that intake of folic acid was nonsignificantly associated with risk for anencephaly (OR = 0.46, CI 95%: [0.25, 0.84]). On the other hand, several studies reported significant relations between low folic acid consumption and NTDs in Italy (4), Iran (7), western Iraq (19), and Austria (21).

Lack of relation between intake of folic acid and NTD can be due to mandatory flour fortification with folic acid in this area which started from June 2006. Our results indicated that rate of NTDs was significantly higher in overweight women compared with normal-weight ones. Several studies showed that being overweight in women was significantly associated with increase of NTDs rate (22-27).

In this study, the mother's education level was not related with NTDs, which was in contrast with other studies in Italia (4) and Turkey (14), reporting significant associations between education level and NTDs. This difference may be due to small sample size in our study. Based on our findings, maternal abortion history was not associated with NTDs. This result was similar to De Marco’s study (4) in Italy, reporting that previous history of spontaneous abortions did not prove to be a spina bifida risk factor. Our study showed that gravidity was not associated with NTD. In contrast with our study, De Marco et al. (4) in Italy showed that birth order was a significant risk with two to three folds higher risk, if the index case was second-birth (OR = 2.15, 95% CI: 1.25–3.69) or third-birth (OR = 3.93, 95% CI: 1.69–9.17), respectively.

Regarding various populations in this area, survey of nutritional factors was the strong point, and lack of all types of hospitals in our province was the limitation of this study. Concerning the high rate of NTDs in this region and due to multifactorial causes of NTDs, this study indicated that maternal overweight, ethnicity and nutrition, may be effective in predisposition to NTDs.
